# Nocturnal heat exposure and stroke risk

**DOI:** 10.1093/eurheartj/ehae277

**Published:** 2024-05-21

**Authors:** Cheng He, Susanne Breitner, Siqi Zhang, Veronika Huber, Markus Naumann, Claudia Traidl-Hoffmann, Gertrud Hammel, Annette Peters, Michael Ertl, Alexandra Schneider

**Affiliations:** Institute of Epidemiology, Helmholtz Zentrum München—German Research Center for Environmental Health (GmbH), Ingolstädter Landstraße 1, D-85764 Neuherberg, Germany; Institute of Epidemiology, Helmholtz Zentrum München—German Research Center for Environmental Health (GmbH), Ingolstädter Landstraße 1, D-85764 Neuherberg, Germany; Institute for Medical Information Processing, Biometry, and Epidemiology, IBE, Medical Faculty, Ludwig-Maximilians-Universität München, Munich, Germany; Institute of Epidemiology, Helmholtz Zentrum München—German Research Center for Environmental Health (GmbH), Ingolstädter Landstraße 1, D-85764 Neuherberg, Germany; Institute of Epidemiology, Helmholtz Zentrum München—German Research Center for Environmental Health (GmbH), Ingolstädter Landstraße 1, D-85764 Neuherberg, Germany; Institute for Medical Information Processing, Biometry, and Epidemiology, IBE, Medical Faculty, Ludwig-Maximilians-Universität München, Munich, Germany; Department of Neurology and Clinical Neurophysiology, University Hospital Augsburg, Augsburg, Germany; Environmental Medicine, Medical Faculty, University Augsburg, Augsburg, Germany; CK-CARE, Christine Kühne Center for Allergy and Research and Education, Davos, Switzerland; Institute of Environmental Medicine, Helmholtz Zentrum München—German Research Center for Environmental Health, Augsburg, Germany; Environmental Medicine, Medical Faculty, University Augsburg, Augsburg, Germany; Institute of Environmental Medicine, Helmholtz Zentrum München—German Research Center for Environmental Health, Augsburg, Germany; Institute of Epidemiology, Helmholtz Zentrum München—German Research Center for Environmental Health (GmbH), Ingolstädter Landstraße 1, D-85764 Neuherberg, Germany; Institute for Medical Information Processing, Biometry, and Epidemiology, IBE, Medical Faculty, Ludwig-Maximilians-Universität München, Munich, Germany; Munich Heart Alliance, German Center for Cardiovascular Health (DZHK e.V., partner-site Munich), Munich, Germany; Department of Neurology and Clinical Neurophysiology, University Hospital Augsburg, Augsburg, Germany; Institute of Epidemiology, Helmholtz Zentrum München—German Research Center for Environmental Health (GmbH), Ingolstädter Landstraße 1, D-85764 Neuherberg, Germany

**Keywords:** Stroke, Ischaemic strokes, Hot night, Climate change

## Abstract

**Background and Aims:**

In recent decades, nighttime temperatures have increased faster than daytime temperatures. The increasing prevalence of nocturnal heat exposure may pose a significant risk to cardiovascular health. This study investigated the association between nighttime heat exposure and stroke risk in the region of Augsburg, Germany, and examined its temporal variations over 15 years.

**Methods:**

Hourly meteorological parameters, including mean temperature, relative humidity, and barometric pressure, were acquired from a local meteorological station. A data set was obtained consisting of 11 037 clinical stroke cases diagnosed during warmer months (May to October) between the years 2006 and 2020. The average age of cases was 71.3 years. Among these cases, 642 were identified as haemorrhagic strokes, 7430 were classified as ischaemic strokes, and 2947 were transient ischaemic attacks. A time-stratified case-crossover analysis with a distributed lag non-linear model was used to estimate the stroke risk associated with extreme nighttime heat, as measured by the hot night excess (HNE) index after controlling for the potential confounding effects of daily maximum temperature and other climatic variables. Subgroup analyses by age group, sex, stroke subtype, and stroke severity were performed to identify variations in susceptibility to nighttime heat.

**Results:**

Results suggested a significant increase in stroke risk on days with extreme nighttime heat (97.5% percentile of HNE) (odds ratio 1.07, 95% confidence interval 1.01–1.15) during the full study period. When comparing the results for 2013–20 with the results for 2006–12, there was a significant increase (*P* < .05) in HNE-related risk for all strokes and specifically for ischaemic strokes during the more recent period. Furthermore, older individuals, females, and patients with mild stroke symptoms exhibited a significantly increased vulnerability to nighttime heat.

**Conclusions:**

This study found nocturnal heat exposure to be related to elevated stroke risk after controlling for maximum daytime temperature, with increasing susceptibility between 2006 and 2020. These results underscore the importance of considering nocturnal heat as a critical trigger of stroke events in a warming climate.

## Structured Graphical Abstract

**Structured Graphical Abstract:**
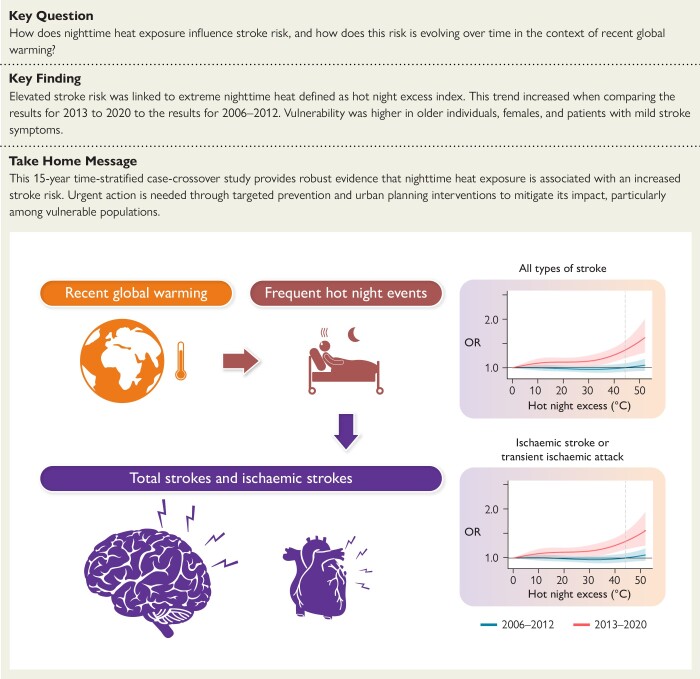
This 15-year time-stratified case-crossover study provides robust evidence that nighttime heat exposure is associated with increased stroke risk. Urgent action is needed through targeted prevention and urban planning interventions to mitigate its impact, particularly among vulnerable populations. OR, odds ratio.

## Introduction

Stroke is a common and debilitating cardiovascular disease that has a considerable impact on global health, causing the third highest number of healthy life years lost due to illness, disability, and premature death worldwide among all diseases.^1^ Therefore, it is essential to identify the risk factors associated with stroke to develop effective public health interventions. Although individual-level risk factors such as arterial hypertension, hypercholesterolaemia, diabetes, alcohol consumption, and smoking are established risk factors,^2–5^ environmental factors, such as non-optimal ambient temperature, may also play a role in the burden of stroke due to widespread exposure across the general population.^6^

The potential consequences of climate change on human health have fostered research about the impact of high ambient temperatures on stroke risk.^6^ Although the association between heat and increased cardiovascular disease risk has been well documented worldwide,^7,8^ there is currently limited and inconsistent evidence on the effects of heat on stroke, particularly different subtypes of strokes. Additionally, while most studies have focused on the effects of daily average or maximum temperatures, recent research has suggested that increases in daily minimum temperatures may also affect the risk of stroke.^9^ Nighttime heat exposure can interrupt the normal physiology of sleep and circadian thermoregulation, which may increase stroke risk.^10^ Despite confirmation of the impact of nighttime heat on daily mortality,^11^ little research has explored its effect on morbidity, and to date, no study has investigated the effect of nighttime heat exposure on risk of stroke.

Understanding temporal variations in the relationship between ambient temperature and health outcomes has emerged as a crucial issue within efforts to estimate the health impacts of future climate warming.^12^ Numerous studies have indicated a declining trend in heat-related mortality risks over time due to factors such as socioeconomic development,^13,14^ but there is limited understanding of the temporal variations in the health effects of nighttime heat. There is evidence that the urban heat island effect exacerbates nighttime heat exposure, and many projections suggest that nighttime warming will surpass daytime warming in various regions globally by this century.^9,15^ This increased risk of nighttime heat exposure may particularly affect certain vulnerable populations, such as the elderly and individuals with pre-existing health conditions, due to their comparatively weaker immune systems. Furthermore, populations residing in regions with lower rates of air conditioning usage may be at heightened risk during nighttime heat events.

Considering the potential health impacts of rising nighttime temperatures, the stronger increase in nocturnal exposure, and its pronounced effects on sensitive groups, we conducted a time-stratified case-crossover study using a validated, complete, and detailed data set of all cases of stroke in Augsburg, Germany, from 2006 to 2020, to assess the association between nocturnal heat exposure and the occurrence of stroke for different periods. Subgroup analyses by stroke subtype, age group, sex, and stroke severity were performed to identify subpopulations with increased susceptibility to nighttime heat.

## Methods

### Study population

The study population consists of residents of the region of Augsburg admitted as patients to the Department of Neurology at the University Hospital Augsburg, a stand-alone comprehensive stroke care facility responsible for more than 750 000 inhabitants in the region. The Department of Neurology at the University Hospital Augsburg is one of the biggest stroke centres in Germany with ∼2000 stroke admissions per year. For this study, we obtained 22 284 recorded stroke cases from 1 January 2006 to 31 August 2020 from the Department. All included cases were classified by using the International Classification of Diseases 10th Revision (ICD-10) system. This classification was based on the final medical registries, which were further verified by the medical controlling unit of the University Hospital Augsburg and have been previously employed in our prior research.^16^ Based on this information, all cases can be further stratified into three main subtypes. These subtypes include transient ischaemic attacks (TIA, symptoms lasting for a maximum of 24 h) (G45), ischaemic strokes (I63), and haemorrhagic strokes (I60, I61, and I62). In addition, we further combined TIA with ischaemic strokes considering their similar underlying mechanisms involving reduced blood flow to the brain. In addition, all cases were also categorized using the National Institutes of Health Stroke Scale (NIHSS) during first admission to the hospital. The NIHSS is a standardized assessment tool that consists of a numerical scoring system used to evaluate stroke symptom severity and assess and monitor the neurological status of a patient who has suffered a stroke. The NIHSS has already been used in many related studies to evaluate the severity of stroke and the effectiveness of new treatments for stroke.^17^ In addition, some of the cases have available information regarding the Trial of Org 10172 in Acute Stroke Treatment (TOAST). This allows for the classification of these stroke cases into categories as large artery disease, cardioembolism, and small vessel disease.

### Exposure data

Hourly meteorological variables (air temperature, relative humidity, and barometric pressure), daily particulate matter with an aerodynamic diameter < 10 mm (PM_10_), nitrogen dioxide (NO_2_), and daily maximum 8 h average ozone (O_3_) concentrations were obtained from the local meteorological station and air quality monitoring stations in Augsburg. Details of these exposure data are explained in the online supplementary material.

### Extreme heat during the night

In order to quantify the intensity of nocturnal thermal stress, we adopted the hot night excess (HNE) index. Referring to previous studies,^9,11^ HNE was calculated by determining the excess sum of high temperature during the night (unit: °C):


$$\mathrm{HNE}=\overset{n_{j}}{\underset{i}{\sum }}\;(t_{ij}-T_{\mathrm{thr}})\times I_{\mathrm{thr}}(t_{ij}),$$

where $n_{j}$ is the total night hours of day *j*, which is from the beginning of the night at day *j-1* to the end of the night in the morning of day *j*, $t_{ij}$ is the nighttime temperature in the hour *i* at night of the day *j*, $T_{\mathrm{thr}}$ is the temperature threshold of nighttime warming, and $I_{\mathrm{thr}}(t_{ij})$ is calculated as:


$$I_{\mathrm{thr}}(t_{ij})=\{\begin{array}{c} \left(0\;if\;t_{ij}<T_{\mathrm{thr}}\right)\\ \left(1\;if\;t_{ij}>T_{\mathrm{thr}}\right) \end{array}.$$

As suggested in previous research,^9^ we determined the threshold based on the daily minimum temperature to quantify the intensity of the heat during the night. Specifically, we defined this threshold as the 95th percentile of daily minimum temperature during the whole study period, which is 14.6°C. The local sunset and sunrise times on different days were derived from the Astral package (version 2.2) in the Python platform (version 3.8.10).

Within the study period, more than 96% of hot night events occurred during the months of May to October (see Supplementary data online, *Table S1*). Temperatures were lower during November to April, averaging 3.31°C, whereas from May to October, the average temperature increases substantially to 14.14°C. To focus our study on the period when most extreme heat events occur, to mitigate potential influences from cold exposures, and to avoid the substantial temperature disparity induced by indoor heating during the cold season, we limited our analysis to stroke cases occurring during the months of May to October.

### Statistical analyses

The association between HNE and stroke was estimated by an individual-level, time-stratified, case-crossover approach.^18,19^ Specifically, we applied conditional logistic regression models to analyse the potential relationship between extreme heat during the night and stroke occurrence. For each case, the exposure on the day of stroke occurrence (‘case’ day) was compared with exposure on days during the same month and on the same day of the week (‘control’ days). This method controls for long-term time trends and seasonality in underlying stroke rates, time-trend bias from the exposure, and time-invariant confounding,^20^ such as some personal risk factors, including hypertension, diabetes, smoking, alcohol consumption, hypercholesterolaemia, obesity, and cardiovascular history.

To account for the potential non-linear and lagged effects of nighttime extreme heat in the main model, we incorporated a distributed lag non-linear model (DLNM) for HNE. Specifically, we introduced a cross-basis function of daily HNE, which includes a quadratic B-spline with two internal knots placed at the 50th and 90th centiles of daily HNE distributions, as done in previous studies.^9,21^ We also explored the lag response curves with a natural cubic B-spline with an intercept and three internal knots placed at equally spaced values in the log scale, with a maximum lag of up to 7 days (Lag 0–6). Previous studies have indicated that the lag effect would become insignificant within 5 days due to temperature-related effects on short-term,^20,22^ so we extended the lag period to 7 days to account for potential short-term harvesting effect.^23^ To avoid any confounding effect of daytime temperature, we controlled for daily maximum temperature using a cross-basis function with the same specification as for HNE. Our main model also included natural cubic B-splines with three degrees of freedom of relative humidity and barometric pressure and a categorical variable of the holidays to control for their potential confounding effects.^24^ To better interpret the odds ratios (ORs) associated with extreme heat during the night, we chose reference days with non-hot night days (HNE = 0°C) as a reference in the exposure–response curve and reported the ORs of stroke and their 95% confidence intervals (CI) at the given HNE values.

To assess the time-varying effects of HNE values, we conducted separate estimations of the HNE–stroke associations for two distinct periods (2006–12 and 2013–20). We chose these periods due to their similar time durations and the comparable total number of cases, as shown in *Table 1*.

**Table 1:** Summary statistics of stroke cases in Augsburg, Germany, from 2006 to 2020

	Entire period	2006–12	2013–20
No. of cases			
All cases of strokes	11 037	5343	5694
Transient ischaemic attacks	2947 (26.7%)	1514 (28.3%)	1433 (25.2%)
Haemorrhagic strokes	642 (5.8%)	301 (5.6%)	341 (6.0%)
Ischaemic strokes	7430 (67.3%)	3510 (65.7%)	3920 (68.8%)
Not specified strokes	18 (0.2%)	18 (0.4%)	0 (0.0%)
Sex			
Male	3600 (32.6%)	1825 (34.2%)	1775 (31.2%)
Female	4799 (43.5%)	2253 (42.2%)	2546 (44.7%)
Unknown	2638 (23.9%)	1265 (23.6%)	1373 (24.1%)
Age (years), mean (SD)	71.3 (13.2)	71.3 (12.9)	71.3 (13.4)
NIHSS at admission			
Minor stroke (1–4)	3737 (33.9%)	1643 (30.8%)	2094 (36.8%)
Moderate stroke (5–15)	5792 (52.5%)	2469 (46.2%)	3323 (58.4%)
Moderate to severe stroke (16–20)	285 (2.6%)	125 (2.3%)	160 (2.8%)
Severe stroke (21–42)	107 (1.0%)	53 (1.0%)	54 (0.9%)
Unknown	1116 (10.2%)	1053 (19.7%)	63 (1.1%)

The percentile represents the percentage of subgroup cases relative to the total number of cases in that period.

NIHSS, National Institutes of Health Stroke Scale.

We calculated the lag-cumulative stroke risk at the 97.5th percentile of HNE distribution relative to non-hot night days. According to our previous study,^20^ we selected this specific cut-off point in order to avoid problems caused by the small sample sizes at extreme temperature levels. To test the statistically significant difference between the estimated exposure–response curves in these two sub-periods, we adopted the multivariate Wald test; a *P*-value < .05 was considered statistically significant.^25^ For the methodology section regarding the significance testing, please refer to the detailed description provided in the online supplementary material.

We then conducted stratified analyses for HNE effects on all stroke events to examine potential effect modification by sex, age, and the scale of NIHSS score, as well as among cases with available TOAST information, for the two time periods.

Finally, for estimating the number of excess stroke cases related to hot night exposure in two time periods (2006–12 and 2013–20). We used the following equation:


$$NS_{ip}=(RR_{ip}-1)\times Case_{i},$$

in which $Case_{i}$ represents the daily average number of stroke cases during the warm months (May to October), which we obtained from our database for the study area. $RR_{ip}$ is the relative stroke risk due to hot night exposure in the day *i* during the period *p*; considering the low prevalence of stroke among the total population, we assumed that *RRs* could be represented by satisfactory estimates of *ORs*, which were calculated based on the HNE value observed in day *i* and estimated exposure–response function for period *p* from our main analysis.

All analyses were performed with R software, version 4.2.3 (R Foundation for Statistical Computing Vienna, Austria).

### Sensitivity analyses

To test the robustness of our findings, we conducted a series of sensitivity analyses for each step of our analysis, including different thresholds of HNE, the removal of daytime temperature control, an alternative daily mean temperature metric as a confounder, potential modifications in the HNE–stroke associations when additionally adjusting for air pollution, and different settings in the knots for exposure–response in our main analysis model. Please see the detailed information for our sensitivity analyses in the online supplementary material.

## Results

### Stroke case and nighttime heat exposure

After the exclusion of fatal cases to concentrate on the effects of nighttime heat exposure on stroke incidence, along with other case selections as depicted in Supplementary data online, *Figure S1*, our analysis ultimately encompassed 11 037 cases of first hospitalization for each individual that did not result in death during the study period, recorded within the warm periods (May to October) from 2006 to 2020. Across these cases, the mean (standard deviation) age was 71.3 (13.2) years. Among these cases, 2947 were TIA, 7430 were ischaemic strokes, and 642 were haemorrhagic strokes. Most of these stroke cases (52.5%) were categorized as moderate strokes (NIHSS score > 5 and <15)^26^ (*Table 1*). During the two periods, 2006–12 and 2013–20, there are similarities in the total number of stroke cases, with 5343 cases in 2006–12 and 5694 cases in 2013–20. There were also similar numbers of each stroke subtype during the two periods. In the 2006–12 period, there were 301 cases of haemorrhagic stroke and 5024 cases of ischaemic strokes and TIA; in 2013–20, there were 341 cases of haemorrhagic strokes and 5353 cases of ischaemic strokes and TIA. The proportion of moderate strokes increased from 46.2% in 2006–12 to 58.4% in 2013–20 (*Table 1*). As shown in Supplementary data online, *Figure S2*, the daily mean temperature during the warm season experienced a marginal and statistically non-significant rise, from 14.5°C (2006–12) to 14.8°C (2013–20) (*P* > .05). The daily maximum temperature also experienced a minor increase from 19.6°C (2006–12) to 20.3°C (2013–20) (*Table 2*). The frequency of days with extreme nighttime heat increased from 79 days (2006–12) to 82 days (2013–20). Notably, the daily mean HNE showed significant variations (*P* < .05) between these two periods, allowing us to investigate whether changes in exposure conditions had any noticeable impact on the risk of stroke.

**Table 2:** Summary statistics of daily temperature and extreme nighttime heat during the warm months (May to October) calculated by hourly observation data in Augsburg, Germany, from 2006 to 2020

	Entire period		2006–12		2013–20		*P*-value^a^
	Mean (SD)	2.5th–97.5th	Mean (SD)	2.5th–97.5th	Mean (SD)	2.5th–97.5th	
Mean temperature, °C	14.7 (7.1)	11.9–18.4	14.5 (4.8)	11.6–18.1	14.8 (5.4)	11.7–18.5	.31
Maximum temperature, °C	20.1 (5.9)	16.0–24.4	19.6 (6.0)	15.7–23.9	20.3 (5.9)	16.0–24.6	.29
HNE > 0°C^b^, days (%)	81 (12)	75–85	79 (12)	79–85	82 (12)	75–84	
HNE during days HNE > 0°C^a^	12.2 (13.0)	2.0–18.9	12.2 (12.7)	1.9–19.0	12.2 (13.2)	2.1–18.9	.04

HNE, hot night excess.

^a^The significance test was performed through a comparison of the differences between the daily mean temperature, maximum temperature, and HNE series during the periods 2006–12 and 2013–20.

^b^HNE calculated by the excess sum of high temperature during the night. No. days HNE > 0°C is calculated during the warm periods (May to October) for each study year.

### Time-varying association of temperature and stroke occurrences

During the entire study period, we observed a significant increase in stroke risk on days with extreme nighttime heat (97.5% percentile of HNE) for all pooled stroke cases and specifically for ischaemic strokes and TIA but not for the haemorrhagic stroke subtype alone (see Supplementary data online, *Figure S3* and *Table 3*). The lag response pattern indicated that the impact of nighttime heat on stroke was immediate, with significant effects appearing within the first 2 days (Lag 0–1) (see Supplementary data online, *Figure S4*).

**Table 3:** Cumulative odds ratios estimated for daily stroke cases (95% confidence interval) associated with extreme heat exposure during the night [97.5th percentile of hot night excess (HNE) distribution] for the overall study period and the periods 2006–12 and 2013–20

	Entire period	2006–12	2013–20	*P*-value^a^
All cases	1.14 (1.01–1.32)	0.99 (0.91–1.08)	1.33 (1.18–1.50)	.03
Haemorrhagic strokes	1.04 (0.83–1.35)	0.98 (0.85–1.15)	1.21 (1.02, 1.48)	.48
Ischaemic strokes and TIA	1.16 (1.02–1.35)	0.99 (0.91–1.09)	1.32 (1.16–1.49)	.03

TIA, transient ischaemic attack.

^a^Significance test based on the difference between OR estimates in 2006–12 and 2013–20.

**Figure 1:**
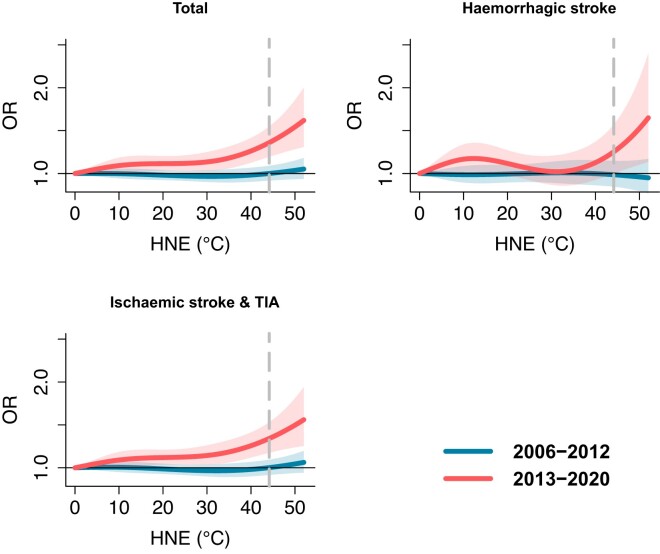
Cumulative exposure–response relationships between hot night excess (HNE) and stroke risk over Lag 0–6 days for 2006–12 and 2013–20 with corresponding 95% confidence intervals (shaded areas). The vertical dashed line represents the 97.5th percentiles of the HNE distribution. Hot night excess represents the excess sum of high temperatures during the night when hourly temperatures are higher than the 95th percentile of daily minimum temperature during the whole study period. For instance, HNE = 0°C indicates non-hot night days during the warm months. HNE = 40°C represents the cumulative sum of hourly nighttime temperatures exceeding the threshold with values accumulating up to 40°C

We found a significant increase in stroke risk associated with nighttime heat exposure over time when comparing the two time periods. The exposure–response curves for all stroke subtypes displayed notable differences (*Figure 1*). During the recent period (2013–20), extreme nighttime heat (97.5th percentile of HNE) exerted significant effects on the risk for all stroke cases and all subtypes, including haemorrhagic and ischaemic stroke. In contrast, there was no significant influence of extreme nighttime heat on all stroke risk, including all subtypes, observed during the earlier period (2006–12). When comparing the results from the time periods 2013–20 to 2006–12 (*Table 2*), the findings highlight a significant increase (*P* < .05) over time in nocturnal thermal stress for all stroke cases and the subgroup comprising ischaemic strokes or TIA. However, it is worth noting that no significant change was observed for haemorrhagic strokes, which may be attributed to the limited number of cases in this category.

In addition, as shown in Supplementary data online, *Figure S5*, by using a consistent and same methodology, we also observed an increase in the impact of daytime temperature (*T*_max_) after controlling the effect of HNE. Additionally, at the same 97.5th percentile, the OR for HNE was slightly higher than for *T*_max_, although this difference did not achieve statistical significance.

The estimation results indicate that from 2006 to 2012, hot nights were attributed to an annual excess of two (95% CI −14–12) cases. However, from 2013 to 2020, hot nights were associated with a total of 33 (95% CI 9–60) excess cases annually among the residents in the study area.

### Subgroup analyses

**Figure 2:**
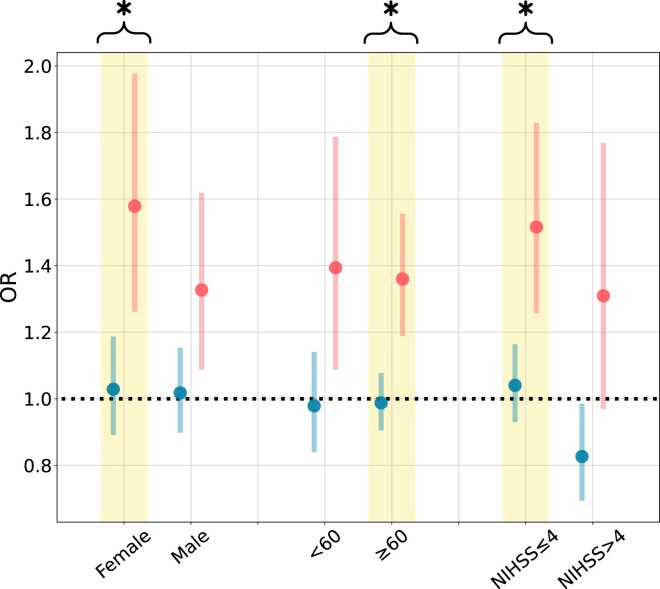
Cumulative odd ratio estimated for daily stroke cases (95% confidence interval) associated with extreme heat exposure during the night [97.5th percentile of hot night excess (HNE) distribution] predicted for 2006–12 and 2013–20 stratified by three different subgroups. Asterisks and coloured groups indicate statistical significance for differences in odd ratio estimates between 2006–12 and 2013–20 (*P* < .05)

Results of the subgroup analyses point to a significant rise in the risk of stroke associated with HNE within specific subpopulations over the study period. *Figure 2* highlights a notable increase in stroke risk for female individuals, older adults (>60 years), and patients diagnosed with minor strokes (NIHSS ≤ 4) from 2006–12 to 2013–20. Specifically, the risk of HNE-related stroke increased from 1.02 (95% CI 0.90–1.15) to 1.33 (95% CI 1.09–1.62) in females, 0.99 (95% CI 0.91–1.08) to 1.36 (95% CI 1.19–1.56) in older adults, and 1.04 (95% CI 0.93–1.16) to 1.52 (95% CI 1.26–1.83) in patients with minor strokes.

During the more recent period, significant HNE-related stroke risks were also found for males and younger age groups. In contrast, there were no significant effects detected for these subgroups from 2006 to 2012.

Furthermore, analysis of cases with available TOAST information revealed that nighttime temperatures significantly increased the risk of strokes due to small vessel disease during 2013–20 compared with the period of 2006–12 (see Supplementary data online, *Table S2*). Additionally, during the 2013–20 phase, we observed a significant impact on strokes caused by cardioembolism (see Supplementary data online, *Table S2*). However, no significant influence was detected for strokes related to large artery disease.

### Sensitivity analyses

As shown in Supplementary data online, *Table S3* and *Figure S6*, the results of the sensitivity analyses consistently revealed an increased risk of stroke when comparing the estimations between the periods 2006–12 and 2013–20, particularly when alternative thresholds (specifically, the 97.5th percentile of daily minimum temperature) were utilized for calculating HNE. As illustrated in Supplementary data online, *Table S4* and *Figures S7–S9*, it is noteworthy that while the influence of HNE on all stroke cases lost significance during the period 2006–12 when adjusting for PM_10_, the impact remained statistically significant during 2013–20, despite small changes in effect size that occurred after controlling for the other three types of pollutants. The observed distinctions between the two periods continued to be statistically significant (*P* < .05) after accounting for various pollutants.

As shown in Supplementary data online, *Table S5* and *Figures S10* and *S11*, the main analysis model also showed no significant changes for the evaluated HNE–stroke associations after removing daily maximum temperature as a covariate or controlling for daily mean temperature instead of daily maximum temperature. The adjustments of knots for the exposure–response functions also did not significantly affect our main results (see Supplementary data online, *Table S5*).

## Discussion

This time-stratified case-crossover study over 15 years showed that nocturnal heat exposure was related to an increased stroke risk after controlling for daytime temperature. We also found that this risk increased during the recent period (2013–20) compared with an earlier period (2006–12). In addition to the effects on all strokes that we observed, we also found effects for ischaemic and haemorrhagic stroke subtypes during the more recent period. Older adults, women, or patients diagnosed with minor strokes (NIHSS < 5), small vessel disease, or cardioembolism experienced a significant increase in nighttime heat-related stroke risk over time (*Structured Graphical Abstract*). These findings suggest that exposure to nighttime heat should be considered a potentially preventable trigger of stroke events under a warming climate, especially given that the intensity of future warming at night is projected to be significantly higher than increases in daytime temperature.

Our results are aligned with existing evidence that nighttime heat can induce an extra health impact in addition to the daily temperature.^9,11^ Specifically, we found that nighttime heat significantly increases the odds of stroke occurrence, suggesting that it may affect health outcomes other than total mortality. This finding is supported by numerous studies that have shown a significant association between heat exposure and increased stroke-related mortality.^7,27^ Our study expands on this knowledge by identifying the specific impact of nighttime heat on stroke occurrence and highlights a clear trend of rising risk in more recent years, further emphasizing the urgent need to address this issue through public policy.

The increased risk of morbidity due to ambient heat detected in this study is in line with our previous finding,^20^ which suggested that population susceptibility to heat-related myocardial infarction increased in Augsburg between the time periods 1987–2000 and 2001–14. There is substantial evidence of increasing tolerance to ambient heat in many parts of the world which may be due to many aspects of adaptation, such as physiological change and behavioural changes like air conditioning usage.^14^ This inconsistency can be explained in a few ways. First, although the average daily mean temperature increases in Augsburg were mild (from 14.5°C during 2003–12 to 14.8°C during 2013–20), the number of days with high temperatures at night and the average HNE was much increased in the recent period. It is also important to note that in Germany, residential air conditioning usage is generally limited and average of usage rates across country lower than 20%.^28^ In addition, according to the German Environment Agency, only ∼4.7% of households in Germany used air conditioning in their living space in 2017. Consequently, people may become more vulnerable to increased heat exposure, especially at night. Additionally, changes in population-level socioeconomic status may also modify that population’s HNE-related stroke risk over time.^20^ In summary, changes in underlying drivers from climatic factors, stroke risk factors, and socioeconomic conditions may contribute to the increased susceptibility to nighttime heat-related stroke over time.

We also found varying results in risk of the stroke subtypes included in this study. We observed that extreme nighttime heat significantly increased the risk of ischaemic strokes and TIA in the full study period, and the magnitude of this risk was higher in the more recent period (2013–20) as compared with the earlier period (2006–12) (*P* < .05). This finding aligns with a recent study indicating a positive association between increased hourly temperature and ischaemic strokes while suggesting that the incidence of haemorrhagic strokes is correlated with low temperatures.^29^ The observed increase in the risk of ischaemic strokes during recent periods can be attributed to factors such as dehydration, elevated blood viscosity, and alterations in blood vessel functioning, which may be exacerbated by the occurrence of more frequent extreme nighttime heat. Specifically, dehydration increases the risk of ischaemic stroke because it concentrates the blood and raises the likelihood of blood clot formation,^30^ especially under the condition of hot weather and with insufficient hydration during the night. Elevated blood viscosity during a hot night increases the risk of ischaemic strokes by promoting blood clot formation, reducing blood flow efficiency, and contributing to hypertension and atherosclerosis.^31^ The potential hypoperfusion in patients with underlying carotid stenosis or intracranial stenosis due to higher nocturnal temperatures may also exacerbate the risk of ischaemic strokes.^32^ The influence of enhanced heat exposure on haemorrhagic strokes appears to be less pronounced, possibly due to the relatively low number of cases.^33^ However, high temperatures can increase the risk of hypertension,^34^ which is one of the risk factors for haemorrhagic stroke.^35^

Differences in risk within population subgroups may be due to differences in physiological characteristics. Increased risk among older individuals as compared with younger individuals can be explained by age-related physiological changes and decreased heat tolerance. These factors also make older individuals especially vulnerable during increasingly frequent of hot night events. In comparison with males, females face an increased risk of nighttime heat-related stroke, which may be explained by hormonal influences and physiological differences. Fluctuations in hormone levels during menstruation and menopause can lower women’s heat tolerance.^36^ Furthermore, women generally possess a higher body fat percentage than men, which reduces their heat dissipation capacity.^37^ Overall, these results suggested that the frequency of hot night events during the recent period may have a more detrimental effect on certain types of patients or sensitive population groups.

The main strength of the present study is the validated, complete, and detailed registration of all stroke cases over 15 years. This large sample size and long coverage enhance the reliability and generalizability of our findings. Second, the case-crossover design strengthens the validity of the findings and helps to rule out alternative cofounders. Third, we found that the impact of nocturnal heat exposure on stroke risk was greater in certain population groups, including older adults, women, and patients treated with minor strokes (NIHSS < 5). This underscores the importance of targeted prevention efforts and interventions to protect those most at risk.

Our findings carry significant public health implications. Firstly, healthcare systems should prioritize implementing heat health action plans that include targeted messaging and resources to mitigate the risks of nocturnal heat exposure. Secondly, community-level interventions such as providing education on heat-related illness prevention and improving urban green spaces can help protect vulnerable populations. Lastly, policymakers should consider integrating climate adaptation measures into urban planning, including implementing heat-resilient infrastructure and urban greening initiatives to reduce the urban heat island effect and mitigate the impact of rising nighttime temperatures.

Our study also has several limitations. First, our exposure data were obtained from one outdoor monitoring station, which may not be able to accurately represent the temperature exposures of cases living in different regions within the study area. However, this measurement error is likely to be random and might result in an underestimation of effect estimates. Second, indoor temperatures experienced by the cases may differ from outdoor temperatures. However, as the study period was limited to the warm season (May to October), the difference between indoor and outdoor temperatures may be less pronounced compared with the colder months. Third, our results are based on a monocentric study in Augsburg, Germany, and may not apply to other regions with different climatic, demographic, and socioeconomic conditions. Future studies using multicentre stroke registries are warranted to confirm our findings in other areas. In addition, our classification of stroke types relied on ICD-10 codes which, despite validation, may still harbour some inaccuracies and potentially introduce biases. Finally, due to the incompleteness of TOAST classification information, our results were unable to provide complete subgroup analysis results. Nevertheless, our current findings still reveal certain significant influencing mechanisms.

In conclusion, our 15-year registry-based time-stratified case-crossover study provides robust evidence that nocturnal heat exposure is associated with an increased risk of stroke. Moreover, we found a significant increase in this risk during the more recent period (2013–20) compared with the earlier period (2006–12), with significant impacts observed for all strokes and the ischaemic stroke subtypes. Certain population groups, including older adults, women, and patients treated with minor strokes, experienced a higher risk of nighttime heat-related stroke. These findings highlight the urgent need for preventive measures to mitigate the potential impact of nocturnal heat stress on stroke events, particularly in the context of future warming at night, which is projected to be significantly higher than daytime warming.

## Supplementary data

Supplementary data are available at *European Heart Journal* online.

## Supplementary Material

ehae277_Supplementary_Data

## Data Availability

Data were collected under a data sharing agreement and cannot be made publicly available.

## Pre-registered Clinical Trial Number

None supplied.
