# Supplementary material for: Nocturnal heat exposure and stroke risk
Source: Eur Heart J. 2024 May 21;45(24):2158–66. doi: 10.1093/eurheartj/ehae277 (PMC11212822; doi:10.1093/eurheartj/ehae277)
Supplement: ehae277_Supplementary_Data [file ehae277_supplementary_data.docx]

**Supplementary Methods**

**Meteorological and air pollution data**

Hourly data on meteorological variables, including air temperature and relative humidity were obtained from an urban background weather observatory located 5 km south of the city center (Landesamt-für-Umwelt, from 2001 on). This particular site was selected for its meteorological data as it effectively represents the climatic conditions of Augsburg, especially in terms of air temperature fluctuations, which have already been utilized in our previous assessments of temperature-related health risks^1^.

Particulate matter with an aerodynamic diameter <10 μm (PM_10_) and nitrogen dioxide (NO_2_) were continuously measured at an urban background station located 1 km southeast of the city center. This monitoring station was established in 2004 and is considered a representative site of the urban background in Augsburg^2^. Daily 24-hour average meteorological variables, PM_10_, NO_2_, and daily maximum 8-hour average O_3_ concentrations were calculated if at least 75% of the hourly measurements were available. We acquired data from three distinct air pollution monitoring sites within Augsburg's urban area, due to the high collinearity observed among these sites' measurements^3^, we selected the main urban station's data as the primary source for sensitivity analysis, given its comprehensive data record during the study period and its consistency with our previous research findings^1,3^.

**Methods for testing the statistical significance of the difference**

To test the statistical significance of the difference in odds ratios (ORs) between the two time periods over different subgroups, according to a related study^4^, we calculated the z score as:

$$z=\frac{(E_{1}-E_{2})}{\sqrt{{(SE_{1})}^{2}+{(SE_{2})}^{2}}}$$

Where $E_{1}$ and $E_{2}$ are the natural logarithms of the estimated OR, $SE_{1}$ and $SE_{2}$ are their respective standard errors calculated from their estimated 95% CIs.

**Sensitivity analysis**

As we mentioned in the main text, we conducted series of sensitivity analyses of our analysis from several aspects including:

1. Defined threshold of heat during the night

In addition to the threshold used in the main analysis (95^th^ percentile of daily minimum temperature), we used two alternative thresholds to define and calculate the intensity of heat stress during the night, which include the 90^th^ and 97.5^th^ percentile of daily minimum temperature.

1. Confounder effect of daily temperature

In order to control the confounder effects of daytime temperature, we added the daily maximum temperature in the main model. In addition to that, we tried to control the mean temperature, instead of the daily maximum, in the main model to test our finding, referring to the related study ^5^.

1. Confounder effect of air pollution

To identify whether there is a potential effect modification of air pollutants on the estimated HNE-stroke associations, we also added three main types of air pollutants, including PM_10_, O_3,_ and NO_2_, in the main model, separately. To control their potential non-linear and lag effects, we used the cross-basis function with the same model setting of HNE but with 3 days lag.

1. Key parameters in the main model

As suggested by the related temperature effect study ^6^, some settings of the key parameters in our main model may affect the estimate results, such as the knots setting, so, we adjusted the knots for exposure-response accordingly and to see if our findings were still stable.

**Table S1** Summary statistics of daily temperature and extreme nighttime heat during the May to October versus November to April from 2006 to 2020

|  | **May to October**  **(Mean ± SD)** | **November to April**  **(Mean ± SD)** |
| --- | --- | --- |
| **Mean temperature (unit: °C)** | 14.14 ± 5.82 | 3.31 ± 6.52 |
| **No. days HNE > 0 °C*** | 81 ± 11 | 4 ± 1 |
| **Mean HNE during days HNE > 0 °C (unit: °C)** | 12.10 ± 2.55 | 1.99 ± 2.72 |

**Table S2** Cumulative odds ratios were calculated for the association between daily stroke cases with different TOAST classifications and extreme nighttime heat exposure (at the 97.5th percentile of the hot night excess (HNE) distribution) during the periods 2006-2012 and 2013-2020. These calculations were exclusively conducted for stroke cases for which TOAST classification information was available.

|  | **No. cases** | **2006-2020** | **2006-2012** | **2013-2020** | ***P* value** |
| --- | --- | --- | --- | --- | --- |
| **Large artery disease** | 197 | 0.61 (0.30-1.25) | 0.22 (0.07-0.69) | 1.15 (0.30-4.30) |  |
| **Cardioembolism** | 194 | 1.64 (0.97-2.78) | 0.78 (0.34-1.77) | 2.01 (1.02, 2.91) |  |
| **Small vessel disease** | 1167 | 1.30 (0.92-1.84) | 0.78 (0.48-1.18) | 1.84 (1.08-3.15) | <0.05 |
| **Other etiology** | 433 | 1.44 (0.79-2.62) | 1.20 (0.51-2.86) | 1.92 (0.73-5.06) |  |

**Table S3** Cumulative OR estimates for daily stroke cases (95% CI) associated with HNE using different threshold in defining HNE.

|  | Period | 95% percentile of T_min_ | | 97.5% percentile of T_min_ | |
| --- | --- | --- | --- | --- | --- |
|  |  | **OR** | **CIs** | **OR** | **CIs** |
| All cases | **2006-2020** | 1.14 | 1.01-1.32 | 1.19 | 1.03-1.39 |
|  | **2006-2012** | 0.99 | 0.91-1.08 | 1.01 | 0.92-1.11 |
|  | **2013-2020** | 1.33 | 1.18-1.5 | 1.36 | 1.19-1.56 |
| Hemorrhagic stroke | **2006-2020** | 1.04 | 0.83-1.35 | 1.12 | 0.79-1.56 |
|  | **2006-2012** | 0.98 | 0.85-1.15 | 1.03 | 0.87-1.2 |
|  | **2013-2020** | 1.21 | 1.02-1.48 | 1.22 | 1.00-1.50 |
| Ischemic stroke  & TIA | **2006-2020** | 1.16 | 1.02-1.35 | 1.20 | 1.02-1.47 |
|  | **2006-2012** | 0.99 | 0.91-1.09 | 1.01 | 0.91-1.11 |
|  | **2013-2020** | 1.32 | 1.16-1.49 | 1.35 | 1.17-1.55 |

**Table S4** Cumulative OR estimates for daily stroke cases (95% CI) associated with HNE with and without adjustment for influenza of air pollution.

|  |  | **2006-2020** | **2006-2012** | **2013-2020** | ***P* value*** |
| --- | --- | --- | --- | --- | --- |
| **Without adjustment** | **All cases** | 1.14 (1.01-1.32) | 0.99 (0.91-1.08) | 1.33 (1.18-1.50) | <0.05 |
|  | **Hemorrhagic stroke** | 1.04 (0.83-1.35) | 0.98 (0.85-1.15) | 1.21 (1,02-1.48) |  |
|  | **Ischemic stroke & TIA** | 1.16 (1.02-1.35) | 0.99 (0.91-1.09) | 1.32 (1.16-1.49) | <0.05 |
| **Control for PM_10_** | **All cases** | 0.99 (0.91-1.07) | 0.98 (0.89-1.07) | 1.22 (1.07-1.39) | <0.05 |
|  | **Hemorrhagic stroke** | 0.94 (0.81-1.08) | 0.95 (0.82-1.11) | 1.07 (0.85-1.35) |  |
|  | **Ischemic stroke & TIA** | 0.99 (0.91-1.08) | 0.98 (0.89-1.07) | 1.21 (1.06-1.38) | <0.05 |
| **Control for O_3_** | **All cases** | 1.12 (1.02-1.23) | 1.08 (0.95-1.23) | 1.32 (1.10-1.58) | <0.05 |
|  | **Hemorrhagic stroke** | 1.06 (0.85-1.33) | 1.06 (0.85-1.33) | 1.29 (1.02-1.62) |  |
|  | **Ischemic stroke & TIA** | 1.13 (1.03-1.24) | 1.15 (1.05-1.26) | 1.33 (1.10-1.6) | <0.05 |
| **Control for NO_2_** | **All cases** | 1.22 (1.12-1.33) | 1.19 (1.08-1.30) | 1.58 (1.38-1.82) | <0.05 |
|  | **Hemorrhagic stroke** | 1.23 (1.06-1.43) | 1.25 (1.06-1.47) | 1.46 (1.14-1.87) |  |
|  | **Ischemic stroke & TIA** | 1.23 (1.11-1.36) | 1.20 (1.09-1.31) | 1.59 (1.38-1.83) | <0.05 |

**Table S5** Cumulative OR estimates for daily stroke cases (95% CI) associated with HNE using different model setting, including different knots setting and controling for mean temperautre instead of maximum temperautre.

|  |  | **2006-2020** | **2006-2012** | **2013-2020** |
| --- | --- | --- | --- | --- |
| **Main model** | **All cases** | 1.14 (1.01-1.32) | 0.99 (0.91-1.08) | 1.33 (1.18-1.50) |
|  | **Hemorrhagic stroke** | 1.04 (0.83-1.35) | 0.98 (0.85-1.15) | 1.21 (1.02-1.48) |
|  | **Ischemic stroke & TIA** | 1.16 (1.02-1.35) | 0.99 (0.91-1.09) | 1.32 (1.16-1.49) |
| **Knots for exposure-response: 10th, 50th, and 90th** | **All cases** | 1.19 (1.02-1.41) | 1.04 (0.94-1.14) | 1.53 (1.33-1.76) |
|  | **Hemorrhagic stroke** | 1.11 (0.83-1.48) | 1.00 (0.84-1.19) | 1.35 (1.06-1.72) |
|  | **Ischemic stroke & TIA** | 1.21 (1.02-1.45) | 1.04 (0.94-1.16) | 1.52 (1.32-1.75) |
| **Control for mean temperautre** | **All cases** | 0.98 (0.82-1.17) | 0.90 (0.76-1.06) | 1.34 (1.02-1.76) |
|  | **Hemorrhagic stroke** | 0.82 (0.6-1.12) | 0.89 (0.67-1.19) | 1.01 (0.63-1.63) |
|  | **Ischemic stroke & TIA** | 0.99 (0.83-1.2) | 0.89 (0.75-1.05) | 1.34 (1.01-1.76) |
| **Do not controling daytime temperature** | **All cases** | 1.15 (1.05-1.24) | 0.99 (0.91-1.08) | 1.54 (1.33-1.79) |
|  | **Hemorrhagic stroke** | 1.01 (0.86-1.19) | 0.99 (0.85-1.15) | 1.48 (1.13-1.94) |
|  | **Ischemic stroke & TIA** | 1.04 (0.95-1.15) | 0.98 (0.91-1.09) | 1.51 (1.3-1.76) |


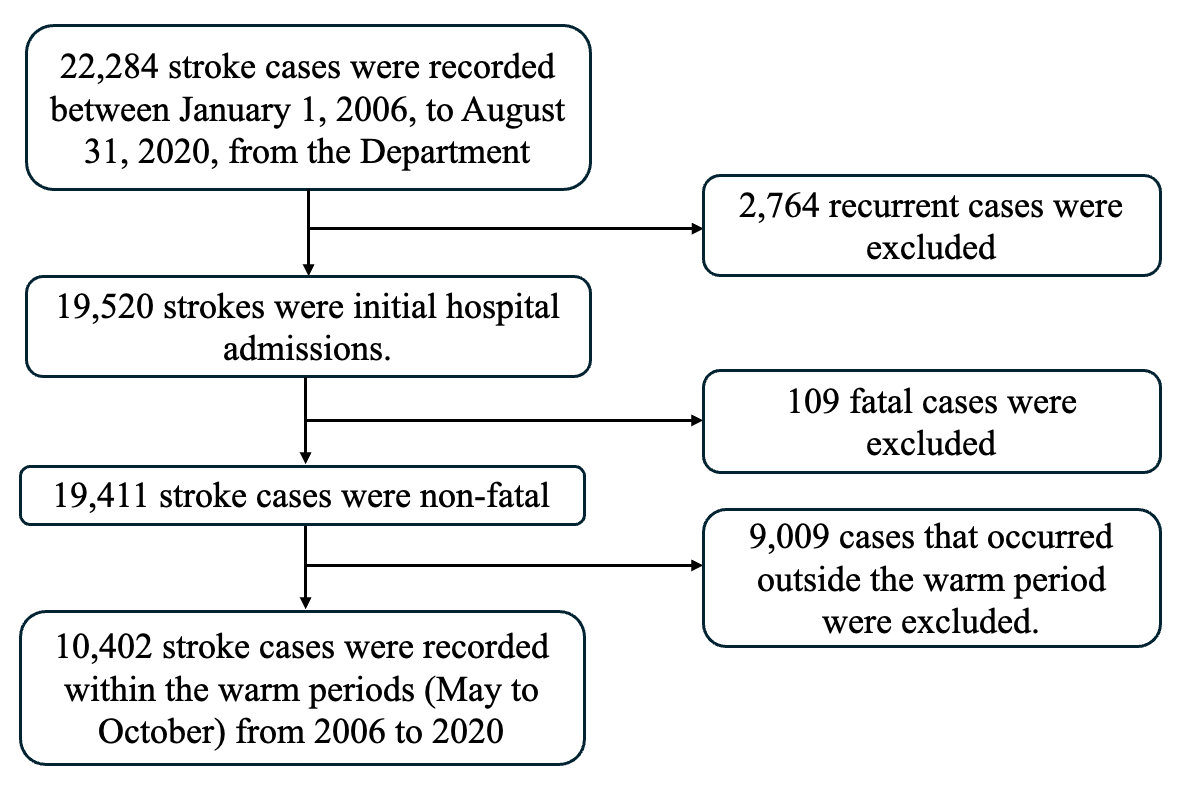


**Figure S1** Road map of case selection


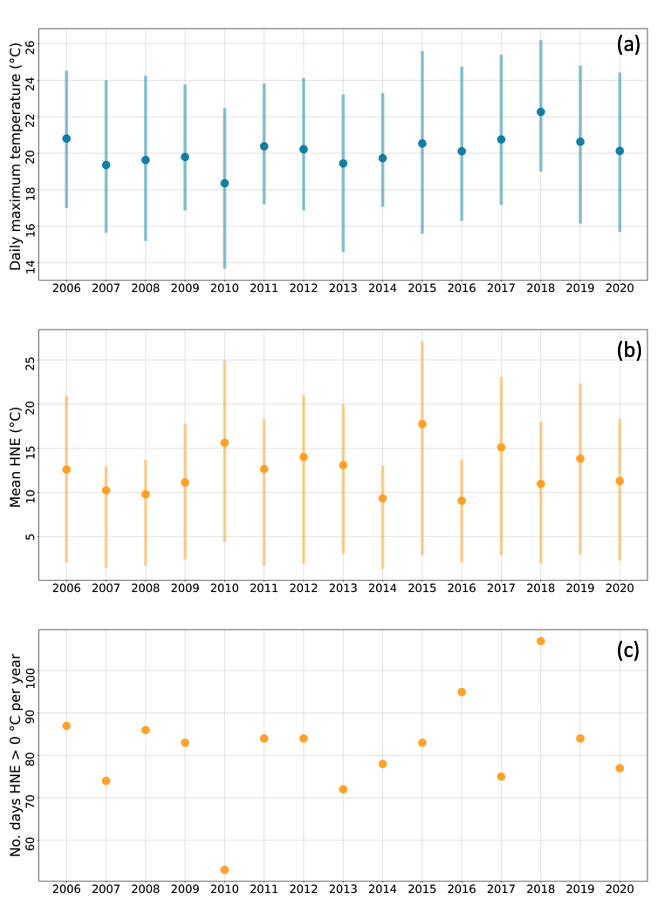


**Figure S2** Daily maximum temperature (a), mean hot night excess (HNE) during the days HNE > 0 °C (b), and number of days HNE > 0 °C per year during the warm months (May to October) in Augsburg, Germany from 2006 to 2020. In the panel a and b, the two ends of the vertical line represent the 25th and 75th percentiles of each year.


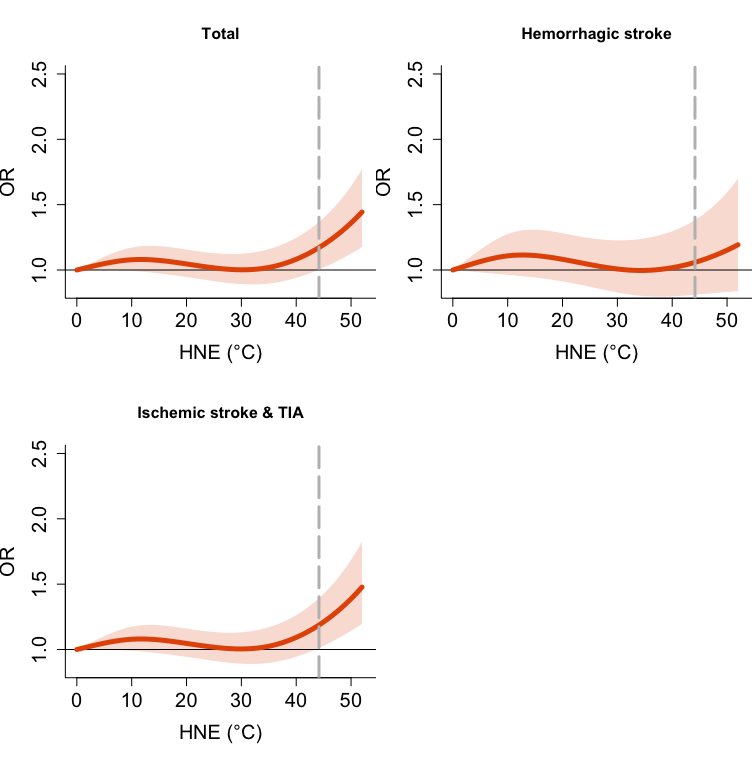


**Figure S3** Cumulative exposure-response relationships between hot night excess (HNE) and stroke risk for the entire period (2006-2020) with a 95% confidence interval. The vertical dashed line represents the 97.5^th^ percentile of the temperature distribution. The solid lines are mean risk estimates, and the shaded areas are their 95% CIs.


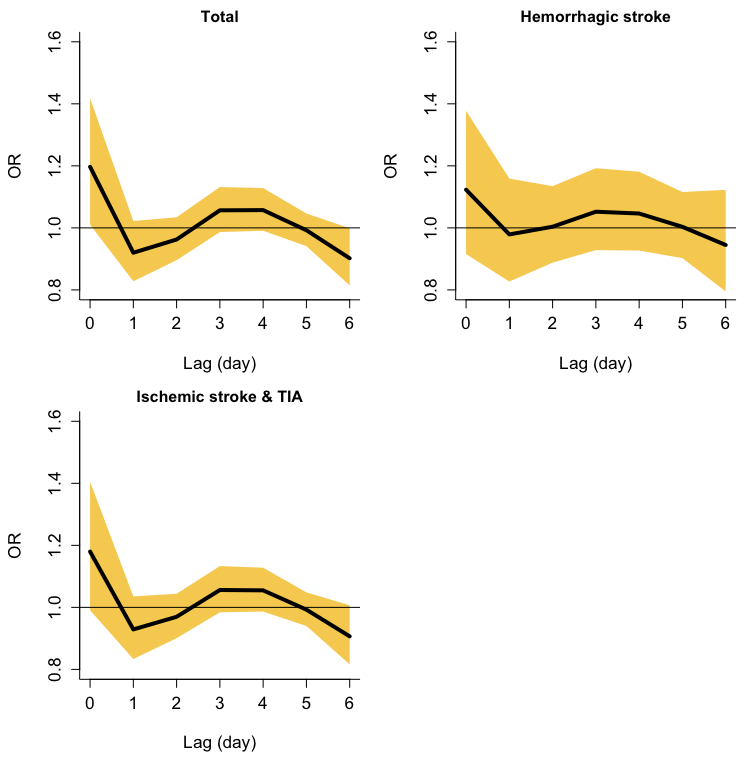


**Figure S4** Lag-response curves for the odds ratio of stroke comparing extreme exposure during the night (the 97.5th percentile of HNE) to the days with non-heat exposure during the night (HNE=0). The solid lines are mean risk estimates, and the shaded areas are their 95% CIs.


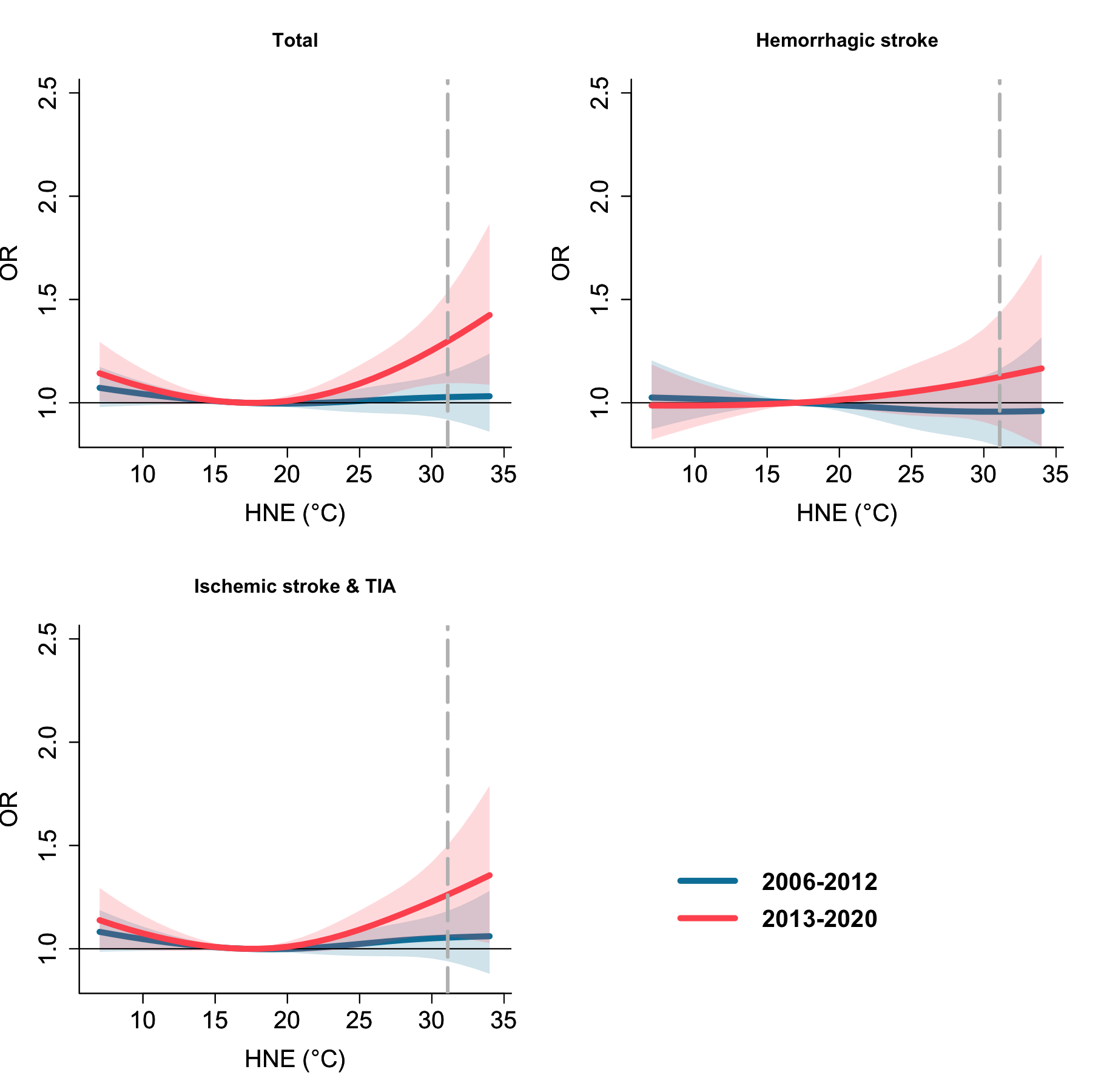


**Figure S5** Cumulative exposure-response relationships between daily maximum temperature and stroke risk over lag 0-6 days for 2006-2012 (blue) and 2013-2020 (red) with corresponding 95% confidence intervals (shaded areas). The vertical dashed line represents the 97.5^th^ percentile of the daily maximum temperatures. We adopted same model setting as we mentioned in the main model for HNE, but we controlled for HNE instead in the main model. We chose the reference day with the minimum stroke ratio temperature during the warm season. The minimum stroke ratio temperature was calculated by scanning through the exposure-response curve estimated from the model to identify the daily maximum temperature value that minimized stroke risk, which is 19.2 based on our daily database.


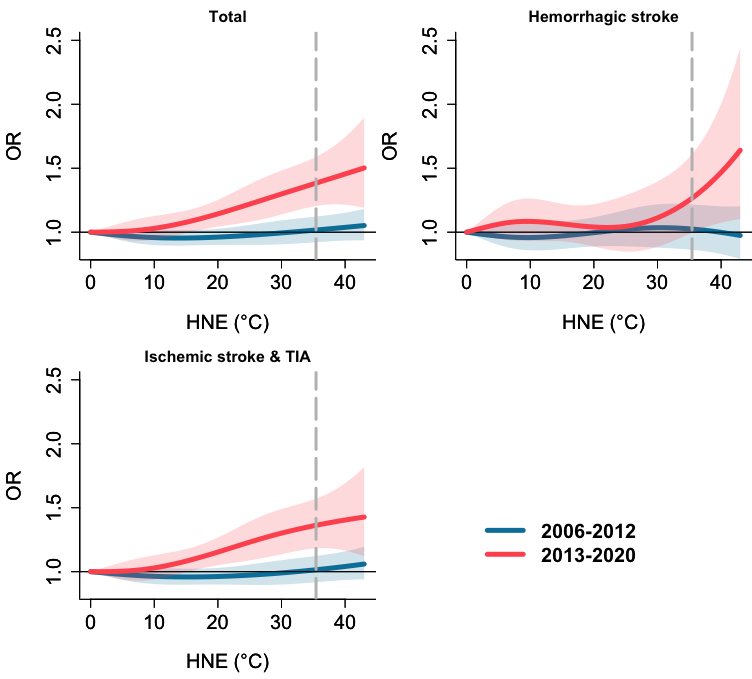


**Figure S6** Cumulative exposure-response relationships between hot night excess, calculated by the 97.5% percentile of T_min_, and stroke risk for 2006-2012 (blue) and 2013-2020 (red) with a 95% confidence interval. The vertical dashed line represents the 97.5th percentile of the temperature distribution.


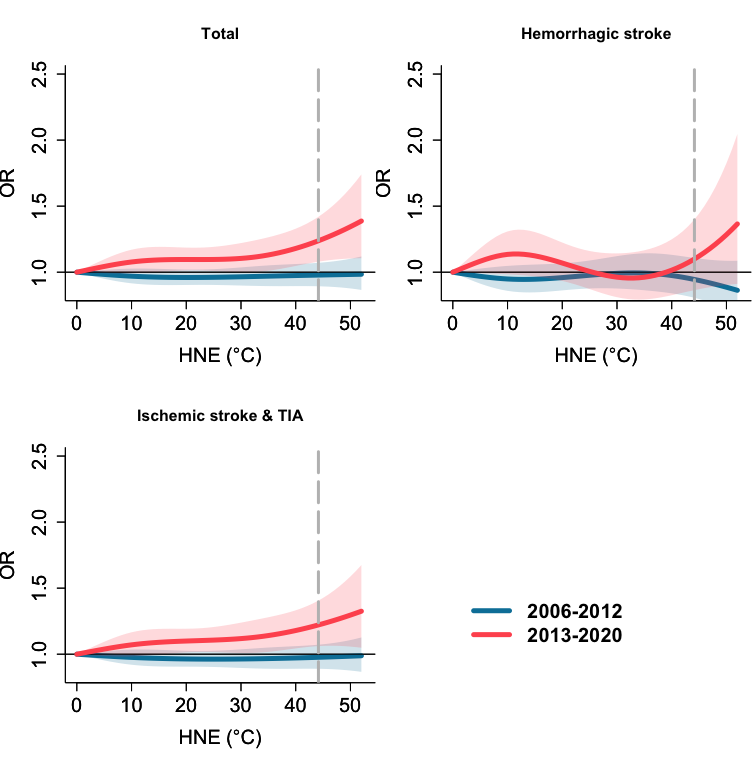


**Figure S7** Cumulative exposure-response relationships between hot night excess and stroke risk for 2006-2012 (blue) and 2013-2020 (red) with a 95% confidence interval after controlling for non-linear and lag effects of daily mean PM_10_. The vertical dashed line represents the 97.5th percentile of the temperature distribution.


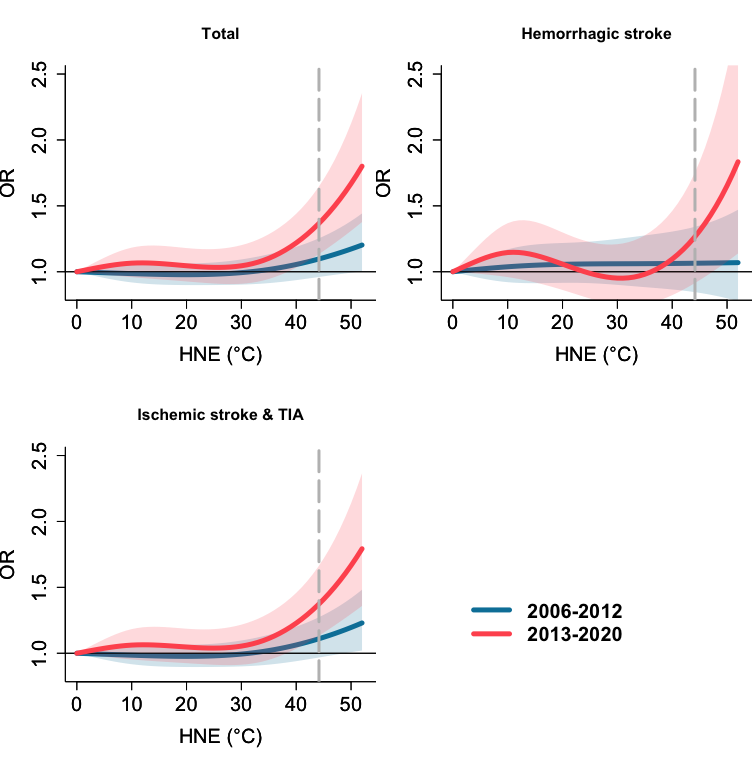


**Figure S8** Cumulative exposure-response relationships between hot night excess and stroke risk for 2006-2012 (blue) and 2013-2020 (red) with 95% confidence interval after controlling for the non-linear and lag effects of daily maximum 8-h moving average O_3_. The vertical dashed line represents the 97.5th percentile of the temperature distribution.


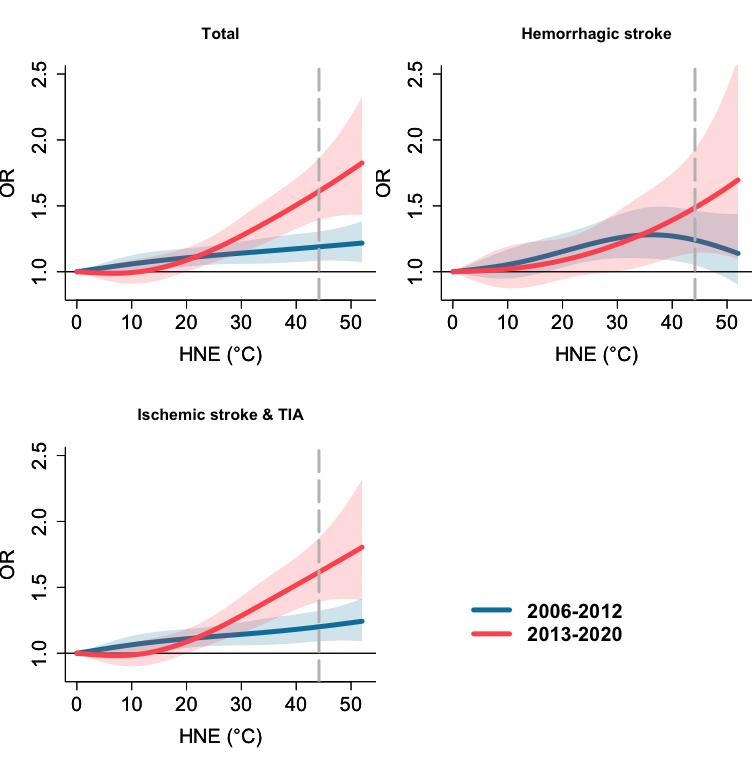


**Figure S9** Cumulative exposure-response relationships between hot night excess and stroke risk for 2006-2012 (blue) and 2013-2020 (red) with a 95% confidence interval after controlling for non-linear and lag effects of daily mean NO_2_. The vertical dashed line represents the 97.5th percentile of the temperature distribution.


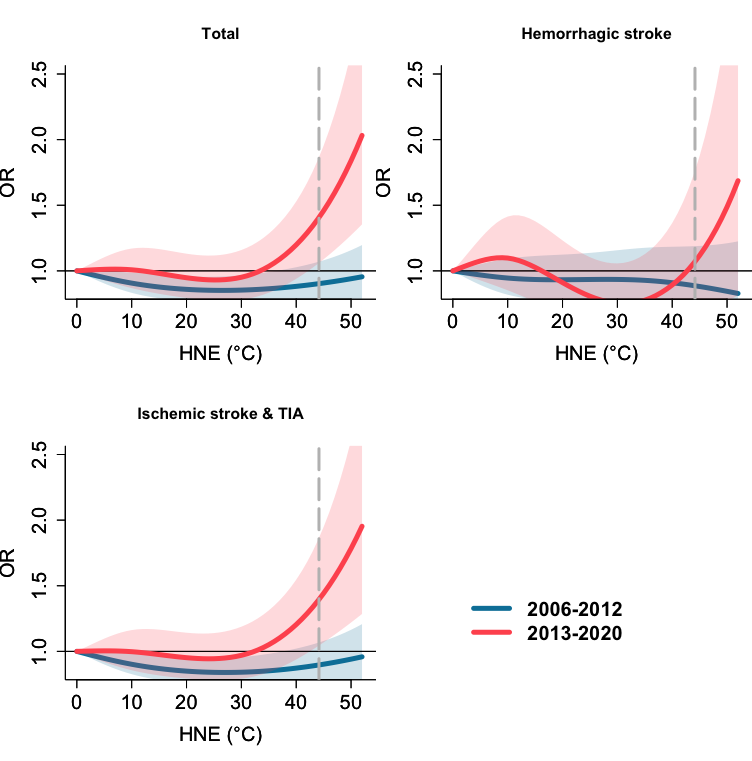


**Figure S10** Cumulative exposure-response relationships between hot night excess and stroke risk for 2006-2012 (blue) and 2013-2020 (red) with a 95% confidence interval, after controlling daily mean temperature instead of daily maximum temperature. The vertical dashed line represents the 97.5th percentile of the temperature distribution.


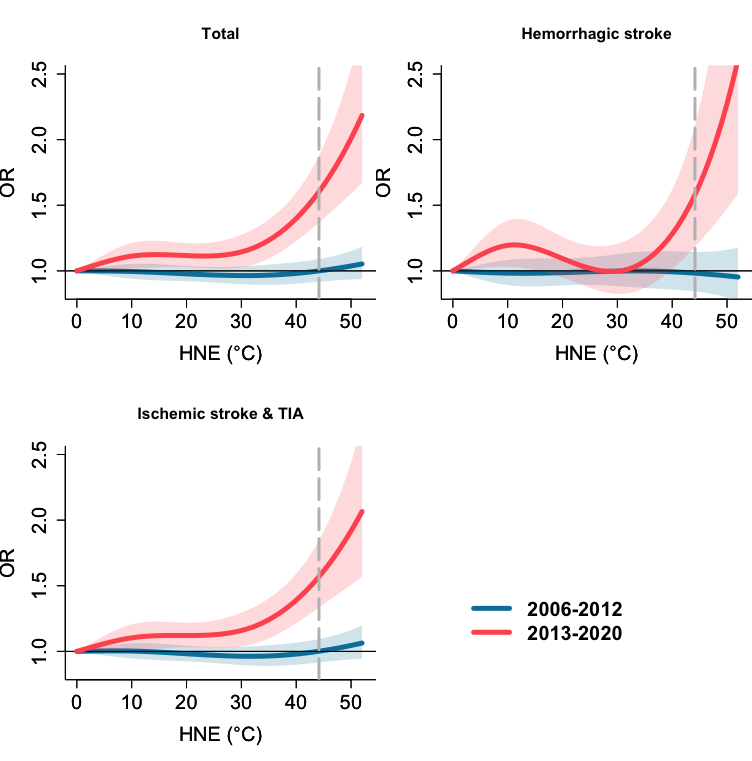


**Figure S11** Cumulative exposure-response relationships between hot night excess and stroke risk for 2006-2012 (blue) and 2013-2020 (red) with a 95% confidence interval, without controlling daily temperature. The vertical dashed line represents the 97.5th percentile of the temperature distribution.

**References**

1. Chen K, Breitner S, Wolf K, Hampel R, Meisinger C, Heier M*, et al.* Temporal variations in the triggering of myocardial infarction by air temperature in Augsburg, Germany, 1987–2014. *European heart journal* 2019;**40**:1600-1608. doi:

2. Cyrys J, Pitz M, Heinrich J, Wichmann H-E, Peters A. Spatial and temporal variation of particle number concentration in Augsburg, Germany. *Science of the Total Environment* 2008;**401**:168-175. doi:

3. Yao Y, Schneider A, Wolf K, Zhang S, Wang-Sattler R, Peters A, Breitner S. Longitudinal associations between metabolites and immediate, short-and medium-term exposure to ambient air pollution: Results from the KORA cohort study. *Science of The Total Environment* 2023;**900**:165780. doi:

4. Chen R, Cai J, Meng X, Kim H, Honda Y, Guo YL*, et al.* Ozone and daily mortality rate in 21 cities of East Asia: how does season modify the association? *American journal of epidemiology* 2014;**180**:729-736. doi:

5. He C, Kim H, Hashizume M, Lee W, Honda Y, Kim SE*, et al.* The effects of night-time warming on mortality burden under future climate change scenarios: a modelling study. *The Lancet Planetary Health* 2022;**6**:e648-e657. doi:

6. Gasparrini A, Guo Y, Hashizume M, Lavigne E, Zanobetti A, Schwartz J*, et al.* Mortality risk attributable to high and low ambient temperature: a multicountry observational study. *The lancet* 2015;**386**:369-375. doi:
